# Who are optimal candidates for primary tumor resection in patients with metastatic gastric adenocarcinoma? A population-based study

**DOI:** 10.1371/journal.pone.0292895

**Published:** 2024-01-24

**Authors:** Xue Song, Yangyang Xie, Yafang Lou

**Affiliations:** 1 Department of Respiratory and Critical Care Medicine, Hangzhou TCM Hospital Affiliated to Zhejiang Chinese Medical University, Hangzhou, Zhejiang Province, China; 2 Department of General Surgery, Hangzhou TCM Hospital Affiliated to Zhejiang Chinese Medical University, Hangzhou, Zhejiang Province, China; Fatebenefratelli Isola Tiberina - Gemelli Isola, ITALY

## Abstract

**Background:**

The research aimed to construct a novel predictive nomogram to identify specific metastatic gastric adenocarcinoma (mGAC) populations who could benefit from primary tumor resection (PTR).

**Method:**

Patients with mGAC were included in the SEER database and divided into PTR and non-PTR groups. The Kaplan-Meier analysis, propensity score matching (PSM), least absolute shrink and selection operator (LASSO) regression, multivariable logistic regression, and multivariate Cox regression methods were then used. Finally, the prediction nomograms were built and tested.

**Results:**

3185 patients with mGAC were enrolled. Among the patients, 679 cases underwent PTR while the other 2506 patients didn’t receive PTR. After PSM, the patients in the PTR group presented longer median overall survival (15.0 vs. 7.0 months, p < 0.001). Among the PTR group, 307 (72.9%) patients obtained longer overall survival than seven months (beneficial group). Then the LASSO logistic regression was performed, and gender, grade, T stage, N stage, pathology, and chemotherapy were included to construct the nomogram. In both the training and validation cohorts, the nomogram exhibited good discrimination (AUC: 0.761 and 0.753, respectively). Furthermore, the other nomogram was constructed to predict 3-, 6-, and 12-month cancer-specific survival based on the variables from the multivariate Cox analysis. The 3-, 6-, and 12-month AUC values were 0.794, 0.739, and 0.698 in the training cohort, and 0.805, 0.759, and 0.695 in the validation cohorts. The calibration curves demonstrated relatively good consistency between the predicted and observed probabilities of survival in two nomograms. The models’ clinical utility was revealed through decision curve analysis.

**Conclusion:**

The benefit nomogram could guide surgeons in decision-making and selecting optimal candidates for PTR among mGAC patients. And the prognostic nomogram presented great prediction ability for these patients.

## Introduction

Gastric cancer (GC) is one of the most common cancers, and it is the third leading cause of cancer-related death globally [1]. Gastric adenocarcinoma (GAC) is the most common subtype of GC [2]. Many GAC cases present with synchronous metastasis upon diagnosis. Unfortunately, patients with metastatic gastric adenocarcinoma (mGAC) tend to suffer poor prognosis, with survival rarely exceeding one year, even in case of receiving up-to-date chemotherapy regimens [3–5]. Currently, these cases are considered medically unsuitable for radical operations.

Palliative gastrectomy and bypass surgery are the primary surgical method in the therapy of mGAC patients. Many studies have indicated that palliative gastrectomy could achieve more satisfactory outcomes in alleviating symptoms such as digestive tract obstruction, tumor-related hemorrhages, and perforation [6–8]. Furthermore, several recent studies demonstrated that palliative gastrectomy could improve quality of life and prolong overall survival [7, 9]. This may attribute to the reduction of severe complications from primary malignancy and potential benefits from multimodal therapy. The value of palliative gastrectomy on non-urgent occasions, however, is unclear. A recent randomized controlled trial (REGATTA) has indicated that metastatic GC patients can’t necessarily benefit from removing the primary tumor [10]. But instead of bringing clarity, the trial generated other controversial results. The limited selection of patients hindered further application in common clinical practice.

Currently, there is no agreement on mGAC therapy, and the value of palliative gastrectomy is still debatable. However, it is evident that some mGAC patients can experience longer survival times following palliative gastrectomy. The need to identify patients who might benefit from palliative gastrectomy is a pressing matter that requires attention.

Using the Surveillance, Epidemiology, and End Results (SEER) database, the retrospective study proposed a useful model to identify specific mGAC groups that could benefit from palliative gastrectomy. Additionally, a prognostic nomogram was created to forecast the survival of these patients.

## Materials and methods

### Ethics statement

The SEER 18 Regs Custom Data Set (2010–2015 dataset) was used for this study. Because the database is open to the public and does not require patient-informed permission, institutional review approval was not necessary for our study. The dataset with the reference number 20317-Nov2021 was extracted.

### Patient selection

**Fig 1:**
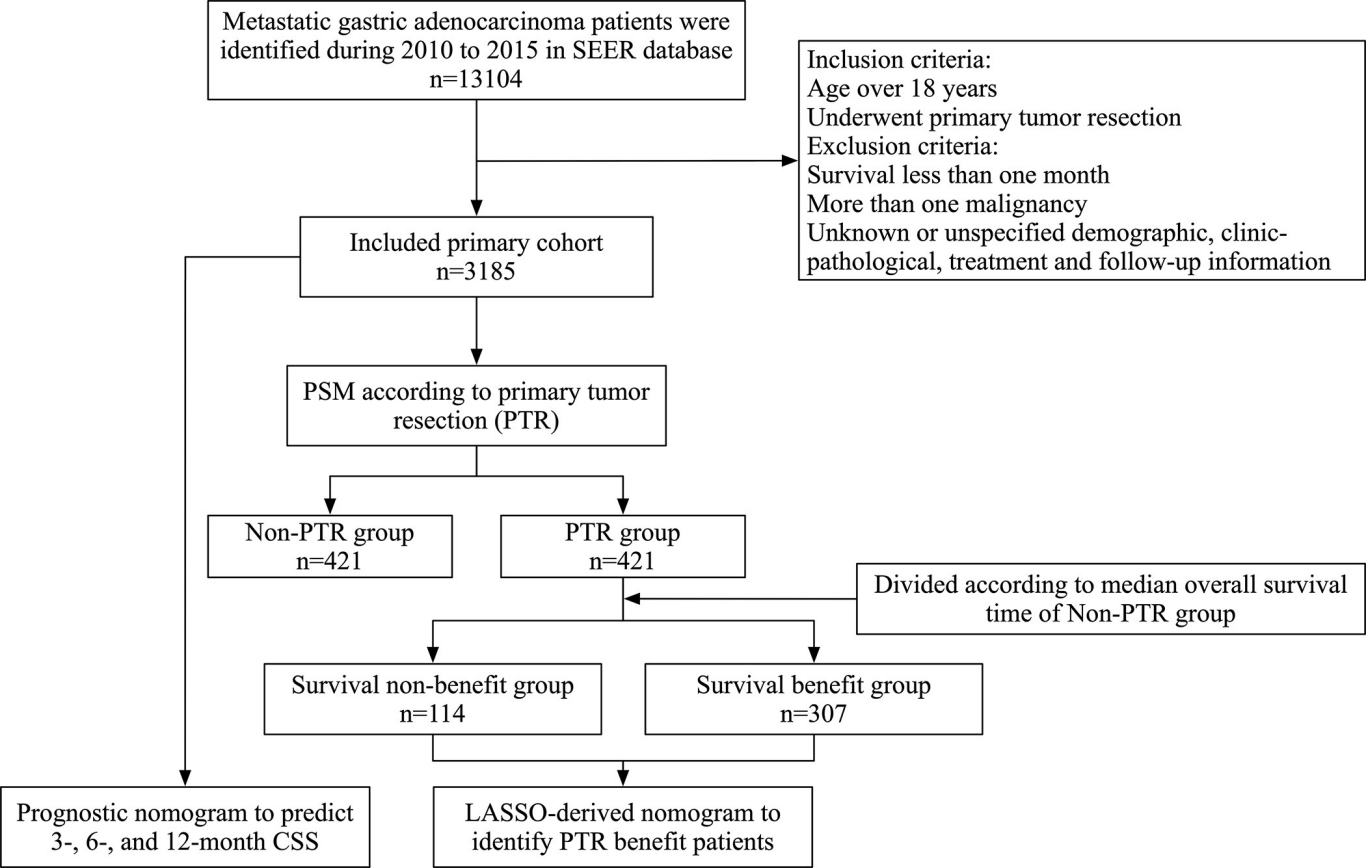
The procedure for patient selection.

The third edition of the International Classification of Diseases for Oncology (ICD-O-3) was used to identify cases of GAC. 8140, 8144, 8145, 8211, 8255, 8260, 8470, 8480, 8481, and 8490 were the histological codes. The inclusion criteria were as follows: (1) older than 18 years old; (2) GAC was the only cancer diagnosis; (3) patients underwent palliative gastrectomy; (4) patients diagnosed with M1 stage. The exclusion criteria were as follows: (1) survival of less than one month; (2) patients with a history of other malignancies; (3) Clinicopathological and follow-up information of patients was not available. Fig 1 depicts the detailed patient selection workflow.

### Clinicopathological variables

Several variables, such as age, gender, race, marital status, grade, T stage, N stage, pathology, primary site, chemotherapy, radiation, year of diagnosis, and prognostic information, were extracted from the database. According to the 8th edition AJCC classification, the patients were reclassified based on the 7^th^ edition recorded in the SEER database [11].

In the study, palliative gastrectomy was classified as primary tumor resection (PTR), which included near-total or total gastrectomy, as well as gastrectomy with resection in conjunction with resection of other organs (SEER RX Summ–Surg Prim Site (1998+), codes 30–33, 40–42, 50–52, 60–63, 80, and 90). The implementation of these surgeries was not related to the location of the tumor. The PTR patients who presented longer OS than the median OS in the non-PTR cohort were regarded as a benefit population.

### Statistical analysis

The baseline clinicopathological characteristics were compared using the chi-square and t-test to determine whether there were any differences. Then, using the univariate Cox proportional hazard model, we conducted subgroup analyses to determine the hazard ratios (HRs) of the two groups in particular patient subgroups. To better illustrate how each parameter affects OS, forest plots were created. We recorded HRs and 95% Confidential Intervals (CIs).

The propensity score matching (PSM) strategy was effective in eliminating confounding bias and imitating randomized controlled trials [12]. The nearest-neighbor algorithm matched cases in both cohorts one-to-one. The parameter alternation was demonstrated using standardized difference (SD). At baseline settings, SD smaller than 0.1 indicated a balanced distribution [13]. The following factors were used for matching in the study: year of diagnosis, age, gender, race, marital status, grade, T stage, N stage, pathology, primary site, chemotherapy, and radiation. The PTR cohort patients were then chosen for further research and randomly assigned to one of two groups: training (70%, n = 295) or validation (30%, n = 126).

Least absolute shrink and selection operator (LASSO) regression, a strategy for identifying separate risk factors, has been shown to significantly reduce redundancy and overfitting in multifactor models [14]. Age, gender, race, marital status, grade, T stage, N stage, pathology, main site, chemotherapy, and radiation were the factors that were taken into account in the LASSO regression. On the training set, a multivariable logistic regression model was created after significant variables were determined through LASSO regression. After that, a predictive nomogram was created to find cases that would benefit from PTR. Each parameter’s "points" line in the model was connected vertically, and the total of each point was equal to the benefit probability. Candidates for PTR benefit were the mGAC cases with benefit probabilities greater than 50%. The patients were then randomly split into two groups: a training group (70%, n = 2230) and a validation group (30%, n = 955). In the training dataset, CSS nomograms for 3-, 6-, and 12-month cancer-specific survival were created using the prognostic features discovered in the multivariate Cox analyses.

The area under the receiver operating characteristic curve (AUC), which measured performance, was initially used to evaluate the two nomograms in the training group before being applied to the validation group. The calibration curves were subjected to 1000 bootstrap resamples to test the consistency of the anticipated and actual survival probability. And the calibration belt was made to evaluate the fit of the model [15]. The receiver operating characteristic (ROC) curves illustrated the capability of the developed model to predict outcomes and compute AUC. Greater prediction power was represented by an AUC with a higher value. By showing net benefit (NB) at a variety of clinically acceptable risk thresholds, decision curve analysis (DCA), a crucial statistical tool, could determine the viability of the nomogram in assisting clinical decisions [16].

All statistical analyses and visualizations were performed using R software (version 4.1.2). In this research, A two-tailed P<0.05 was considered statistically significant.

## Results

### Clinicopathological characteristics

A total of 3185 cases were finally included in the database. Of these patients, 679 cases underwent PTR while the other 2506 patients didn’t receive PTR. Significant differences existed in the characteristics of the two groups, including year at diagnosis, age, gender, race, grade, T stage, N stage, primary site, chemotherapy, and radiation (all p < 0.05). The patients underwent PTR tended to present a higher proportion of 2010–2012 period (55.5% vs. 47.0%) female (40.6% vs. 33.9%), non-white race (35.3% vs. 26.4%), grade III (75.7% vs. 73.5%), grade IV (3.5% vs. 1.1%), T3 stage (33.4% vs. 26.3), T4 stage (56.7% vs. 30.9%), N2 stage (21.8% vs. 7.6%), N3 stage (43.4% vs. 5.1%), distal site (35.9% vs. 14.2%), middle site (26.2% vs. 23.9), no-chemotherapy (34.2% vs. 27.1%), and no-radiotherapy (82.6% vs. 78.9%).

We used 1:1 PSM to decrease the impact of potential confounders due to unmatched parameters across the two cohorts. Following PSM, the SD for most of the variables was less than 0.1, indicating good balancing performance (S1 Fig). 842 cases were ultimately split into cohort PTR (n = 421) and cohort non-PTR (n = 421). Table 1 displayed the baseline characteristics prior to and following PSM.

**Table 1:** The characteristics of mGAC patients before and after PSM.

Characteristics	Before PSM			P value	After PSM			P value
	All	PTR	Non-PTR		All	PTR	Non-PTR	
	N = 3185	N = 679	N = 2506		N = 842	N = 421	N = 421	
Year at diagnosis:				<0.001				0.836
2010–2012	1554 (48.8%)	377 (55.5%)	1177 (47.0%)		420 (49.9%)	208 (49.4%)	212 (50.4%)	
2013–2015	1631 (51.2%)	302 (44.5%)	1329 (53.0%)		422 (50.1%)	213 (50.6%)	209 (49.6%)	
Age	61.9 (14.0)	60.7 (14.4)	62.3 (13.8)	0.01	61.3 (14.2)	61.1 (14.6)	61.6 (13.7)	0.595
Gender:				0.001				0.775
Female	1125 (35.3%)	276 (40.6%)	849 (33.9%)		313 (37.2%)	159 (37.8%)	154 (36.6%)	
Male	2060 (64.7%)	403 (59.4%)	1657 (66.1%)		529 (62.8%)	262 (62.2%)	267 (63.4%)	
Race:				<0.001				0.556
White	2284 (71.7%)	439 (64.7%)	1845 (73.6%)		569 (67.6%)	280 (66.5%)	289 (68.6%)	
Non-White	901 (28.3%)	240 (35.3%)	661 (26.4%)		273 (32.4%)	141 (33.5%)	132 (31.4%)	
Marital status:				0.057				0.664
Married	1990 (62.5%)	446 (65.7%)	1544 (61.6%)		549 (65.2%)	271 (64.4%)	278 (66.0%)	
Unmarried	1195 (37.5%)	233 (34.3%)	962 (38.4%)		293 (34.8%)	150 (35.6%)	143 (34.0%)	
Grade:				<0.001				0.795
I	69 (2.2%)	13 (1.9%)	56 (2.2%)		18 (2.1%)	10 (2.4%)	8 (1.9%)	
II	709 (22.3%)	128 (18.9%)	581 (23.2%)		179 (21.3%)	85 (20.2%)	94 (22.3%)	
III	2356 (74.0%)	514 (75.7%)	1842 (73.5%)		627 (74.5%)	318 (75.5%)	309 (73.4%)	
IV	51 (1.6%)	24 (3.5%)	27 (1.1%)		18 (2.1%)	8 (1.9%)	10 (2.4%)	
T stage:				<0.001				0.262
T1	894 (28.1%)	30 (4.4%)	864 (34.5%)		76 (9.0%)	30 (7.1%)	46 (10.9%)	
T2	245 (7.7%)	37 (5.4%)	208 (8.3%)		62 (7.4%)	31 (7.4%)	31 (7.4%)	
T3	887 (27.8%)	227 (33.4%)	660 (26.3%)		318 (37.8%)	166 (39.4%)	152 (36.1%)	
T4	1159 (36.4%)	385 (56.7%)	774 (30.9%)		386 (45.8%)	194 (46.1%)	192 (45.6%)	
N stage:				<0.001				0.119
N0	1140 (35.8%)	104 (15.3%)	1036 (41.3%)		203 (24.1%)	104 (24.7%)	99 (23.5%)	
N1	1284 (40.3%)	132 (19.4%)	1152 (46.0%)		262 (31.1%)	132 (31.4%)	130 (30.9%)	
N2	339 (10.6%)	148 (21.8%)	191 (7.6%)		207 (24.6%)	113 (26.8%)	94 (22.3%)	
N3	422 (13.2%)	295 (43.4%)	127 (5.1%)		170 (20.2%)	72 (17.1%)	98 (23.3%)	
Pathology:				0.228				0.935
Non-SRCC	2436 (76.5%)	507 (74.7%)	1929 (77.0%)		644 (76.5%)	321 (76.2%)	323 (76.7%)	
SRCC	749 (23.5%)	172 (25.3%)	577 (23.0%)		198 (23.5%)	100 (23.8%)	98 (23.3%)	
Primary site:				<0.001				0.589
Cardia	1065 (33.4%)	94 (13.8%)	971 (38.7%)		195 (23.2%)	93 (22.1%)	102 (24.2%)	
Distal site	600 (18.8%)	244 (35.9%)	356 (14.2%)		219 (26.0%)	118 (28.0%)	101 (24.0%)	
Middle site	778 (24.4%)	178 (26.2%)	600 (23.9%)		212 (25.2%)	105 (24.9%)	107 (25.4%)	
Overlapping/NOS	742 (23.3%)	163 (24.0%)	579 (23.1%)		216 (25.7%)	105 (24.9%)	111 (26.4%)	
Chemotherapy:				<0.001				1
None	911 (28.6%)	232 (34.2%)	679 (27.1%)		253 (30.0%)	127 (30.2%)	126 (29.9%)	
Chemotherapy	2274 (71.4%)	447 (65.8%)	1827 (72.9%)		589 (70.0%)	294 (69.8%)	295 (70.1%)	
Radiation:				0.035				0.67
None	2537 (79.7%)	561 (82.6%)	1976 (78.9%)		668 (79.3%)	337 (80.0%)	331 (78.6%)	
Radiotherapy	648 (20.3%)	118 (17.4%)	530 (21.1%)		174 (20.7%)	84 (20.0%)	90 (21.4%)	

### Survival analysis

**Fig 2:**
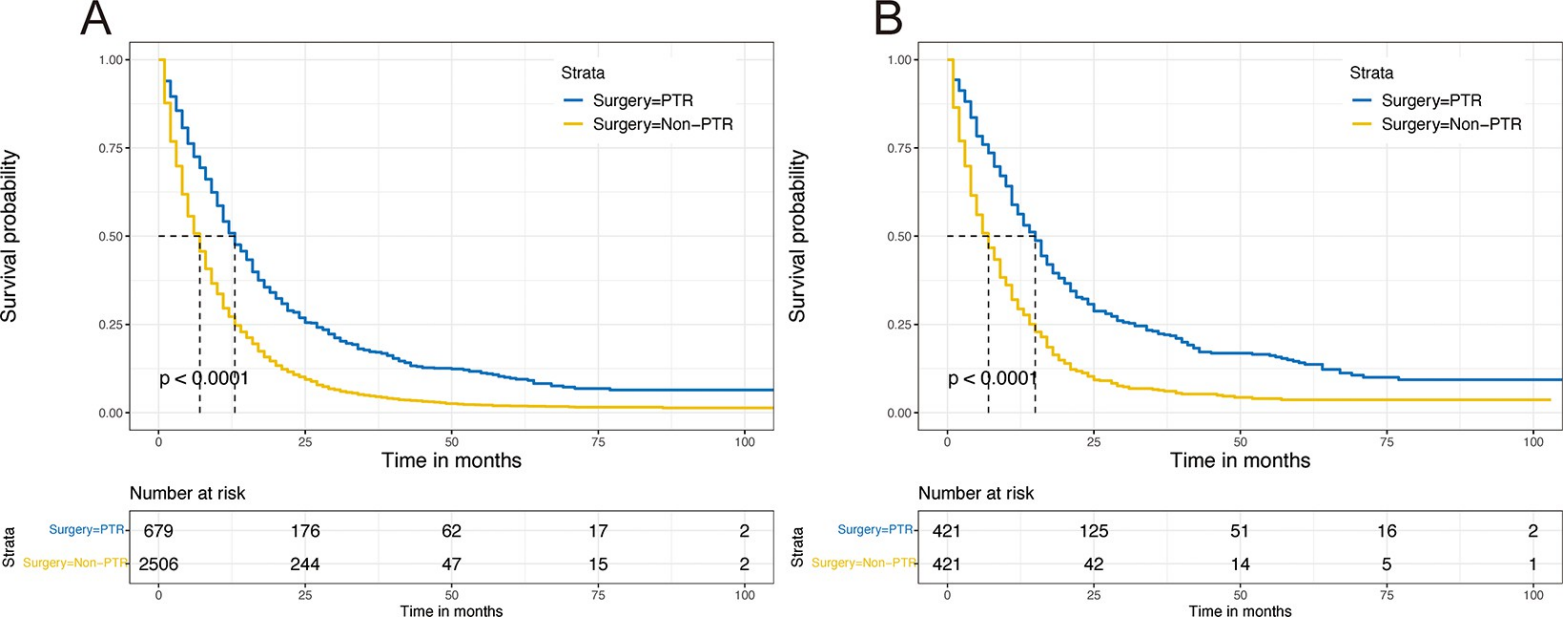
KM survival analysis in the PTR cohort and non-PTR cohort. (A) Before PSM. (B) After PSM.

**Fig 3:**
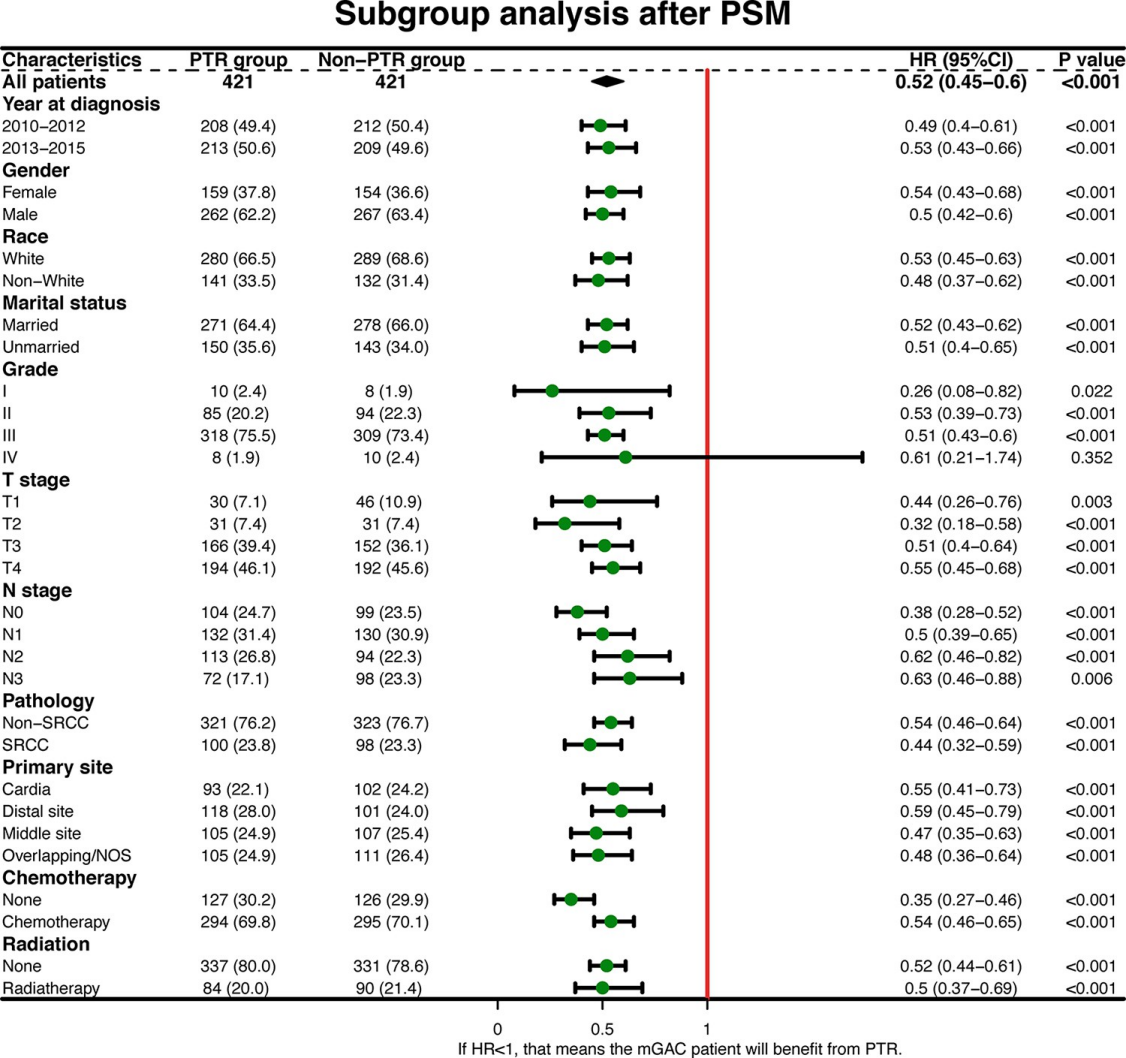
Hazard ratios (HRs) for overall survival after PSM in the two groups are presented in forest plots. Error bars show 95% confidence intervals, while diamonds denote effect size, which was estimated independently in various subgroups.

The Kaplan-Meier analysis revealed a significant difference in OS between the two groups at the pre- and post-match periods. Prior to PSM, patients who underwent PTR had a longer median OS than the non-PTR sample (13.0 vs. 7.0 months, p < 0.001) (Fig 2A). A similar result was obtained after PSM (15.0 vs. 7.0 months, p < 0.001) (Fig 2B). In the forest plots, HRs were less than one in all subgroups except grade IV before and after PSM, showing that PTR could help mGAC patients in most subgroups (Fig 3 and S2 Fig).

### Constructing and verifying the benefit nomogram

**Fig 4:**
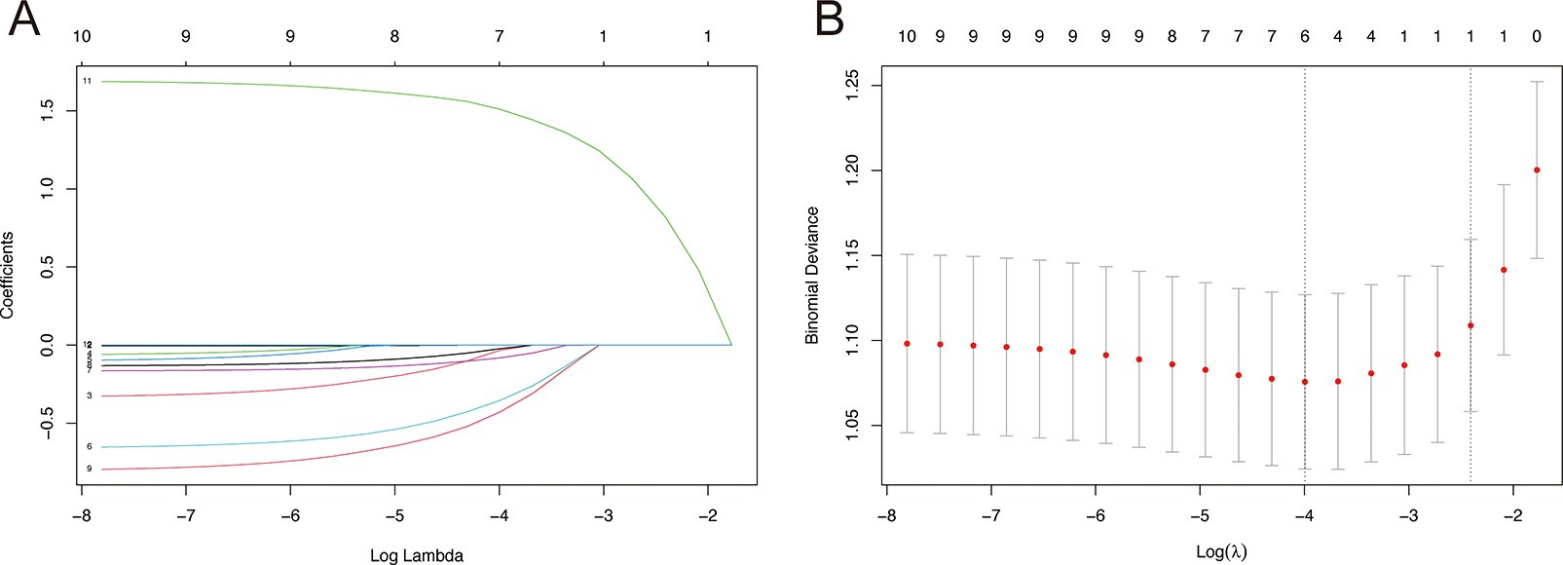
LASSO regression-based feature selection. (A) LASSO coefficient profiles for clinical and pathological characteristics. (B) Fivefold cross-validation is used to choose the tuning parameter (lambda) in the LASSO regression.

For further analysis, the patients from the PTR cohort were split into two groups at random: a training group (70%, n = 295) and a validation group (30%, n = 126). S1 Table displays the primary characteristics of the two groups. To avoid overfitting, a total of 11 variables were included in the LASSO Logistic Regression depending on the training group (Fig 4). The findings identified six independent predictors of the likelihood that mGAC patients undergoing PTR will benefit, including gender, grade, T stage, N stage, pathology, and chemotherapy.

**Fig 5:**
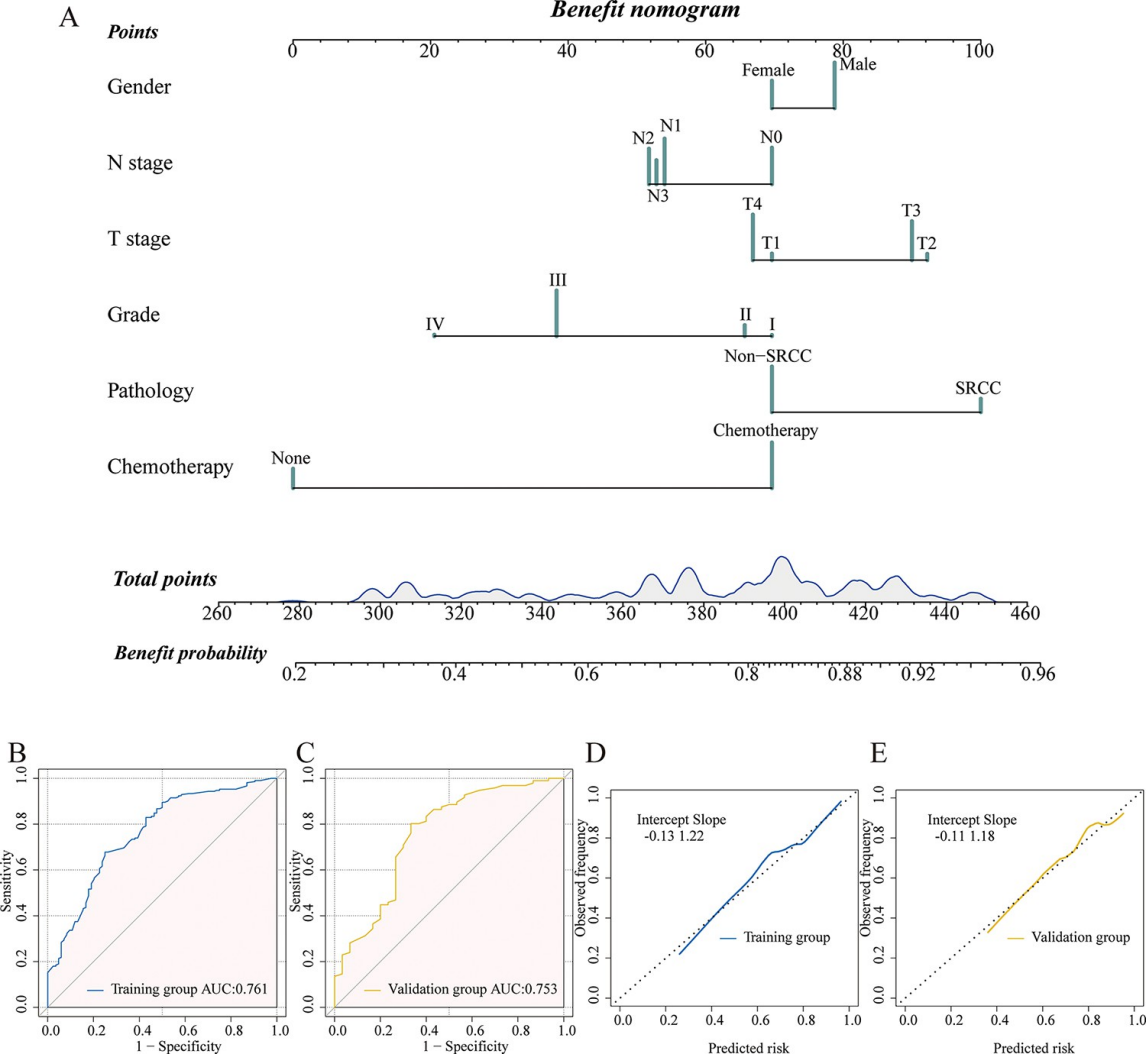
The benefit nomogram to identify optimal candidates among mGAC patients (A). ROC curves in the training (B) and validation (C) set. Calibration curves in the training (D) and validation (E) set.

Then we constructed a predictive nomogram to identify potential PTR-beneficial patients based on the variables from the LASSO Logistic regression (Fig 5A). Clinicians could use the scores corresponding to six parameters to predict the probability of PTR benefit in mGAC patients. The total points were obtained by summing these scores. The model demonstrated strong discriminating power in both the training (AUC = 0.761) and validation (AUC = 0.753) populations, as determined by the AUC index (Fig 5B and 5C). The model’s prediction accuracy was further tested using calibration plots, and the findings showed that the anticipated and actual probabilities were quite consistent (Fig 5D and 5E). Next, DCA curves in both sets (S3A and S3B Fig) confirmed the nomogram’s excellent clinical applicability. And the calibration belt validated the good fit of the model (S4 Fig). Then the subgroup analyses were performed based on different tumor locations. The AUC values were 0.737 and 0.833 in the subgroup of the cardia and distal site (S5 Fig). The aforementioned results demonstrated our nomogram’s strong credibility and good predictive capability.

### Constructing and verifying the prognostic nomogram

**Fig 6:**
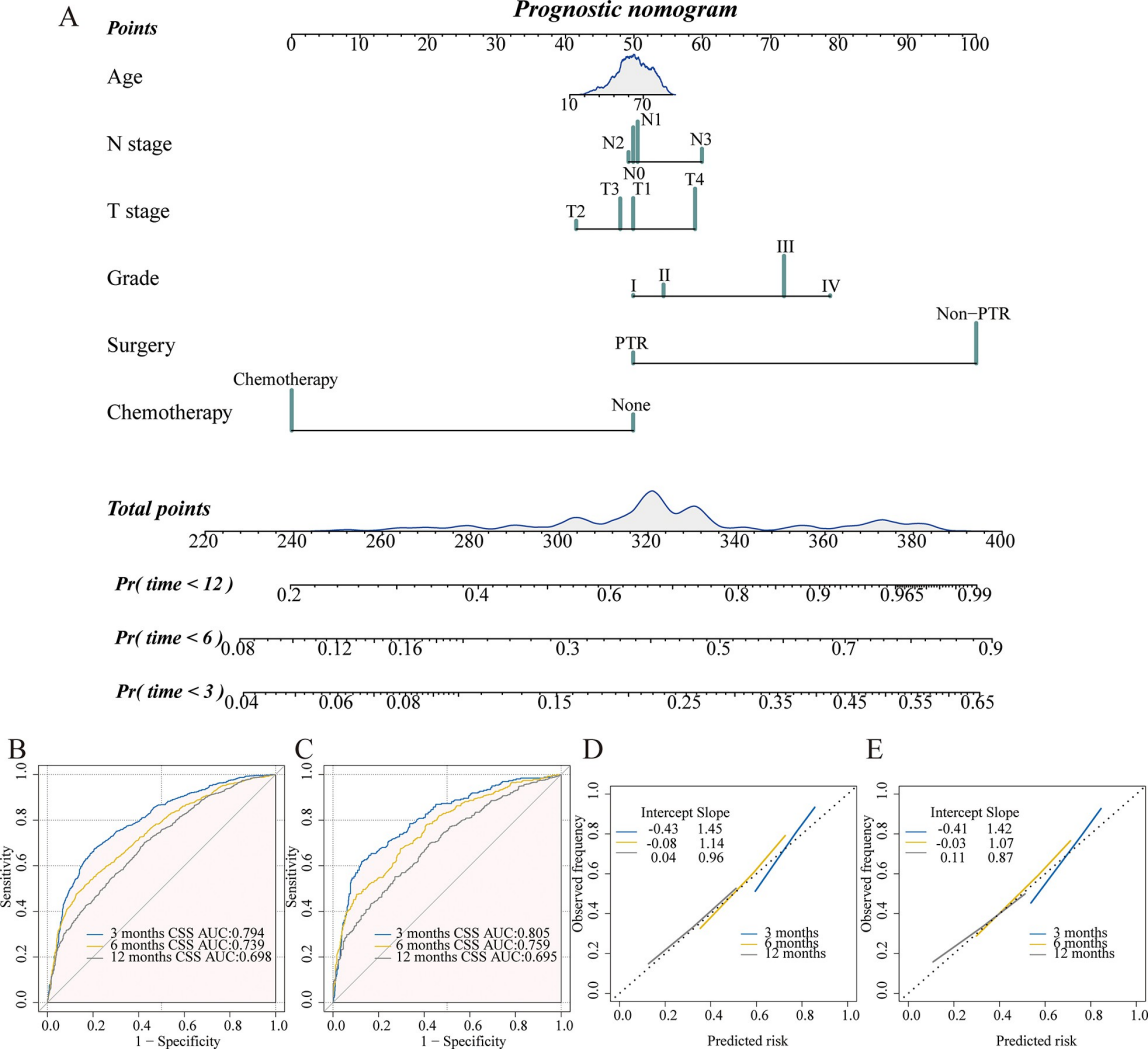
The prognostic nomogram to predict 3-, 6-, and 12-month CSS among mGAC patients (A). ROC curves in the training (B) and validation (C) set. Calibration curves in the training (D) and validation (E) set.

Then, we carried out a separate second analysis. We randomly divided the total patient population into a training group (70%, n = 2230) and a validation group (30%, n = 955) with the goal of creating a nomogram to predict 3-, 6-, and 12-month CSS in mGAC patients. Then, the multivariate Cox regression was used to further identify the significant variables (P < 0.2) from the univariate Cox regression. This revealed that age, grade, T stage, N stage, surgery, and chemotherapy were independent predictors of CSS in mGAC patients (Table 2 and Fig 6A).

**Table 2:** Univariate and multivariate Cox regression analysis of CSS for each variable in mGAC patients in the training cohort.

Characteristics	Univariate analysis			Multivariate analysis		
	HR	95%CI	P	HR	95%CI	P
Age	1.01	1.01–1.01	<0.001	1	1–1.01	0.039
Gender						
Female	Reference					
Male	0.99	0.9–1.08	0.795			
Race						
White	Reference					
Non-White	0.97	0.88–1.06	0.473			
Marital status						
Married	Reference			Reference		
Unmarried	1.13	1.03–1.24	0.008	1.04	0.95–1.13	0.456
Grade						
I	Reference			Reference		
II	1.18	0.86–1.64	0.306	1.09	0.79–1.51	0.603
III	1.45	1.06–1.98	0.021	1.54	1.12–2.11	0.008
IV	1.51	0.95–2.4	0.083	1.76	1.1–2.81	0.018
T stage						
T1	Reference			Reference		
T2	0.71	0.59–0.85	<0.001	0.85	0.71–1.02	0.08
T3	0.78	0.69–0.87	<0.001	0.96	0.85–1.09	0.554
T4	0.95	0.86–1.06	0.404	1.19	1.06–1.34	0.003
N stage						
N0	Reference			Reference		
N1	0.99	0.9–1.09	0.834	1.01	0.92–1.12	0.797
N2	0.78	0.67–0.92	0.002	0.99	0.84–1.17	0.88
N3	0.78	0.68–0.89	<0.001	1.22	1.04–1.43	0.017
Pathology						
Non-SRCC	Reference					
SRCC	1.01	0.92–1.12	0.806			
Primary site						
Cardia	Reference					
Distal site	0.93	0.82–1.05	0.228			
Middle site	1	0.89–1.13	0.967			
Overlapping/NOS	1.04	0.92–1.17	0.549			
Chemotherapy						
None	Reference			Reference		
Chemotherapy	0.46	0.42–0.51	<0.001	0.38	0.34–0.42	<0.001
Radiation						
None	Reference					
Radiotherapy	0.94	0.84–1.05	0.244			
Surgery						
PTR	Reference			Reference		
Non-PTR	1.88	1.68–2.1	<0.001	2.66	2.32–3.04	<0.001

The validation cohort was used to confirm the nomogram that was created using the training cohort. The training cohort’s 3-, 6-, and 12-month AUC values were 0.794, 0.739, and 0.698, while the validation cohorts’ values were 0.805, 0.759, and 0.695 (Fig 6B and 6C). High AUC values demonstrated strong discrimination abilities. Additionally, we tested the model’s predictive power using calibration plots, which showed a generally consistent relationship between the anticipated and actual survival rates (Fig 6D and 6E). Next, DCA curves in both sets showed the nomogram’s excellent clinical practical applicability (S3C-S3H Fig). The results demonstrated our nomogram’s strong credibility and good predictive capability.

## Discussion

According to this study, mGAC patients who had PTR had longer survival times than those who did not. For identifying the subgroup that would profit from PTR, a predictive nomogram was created. The nomogram that included information on the patient’s gender, grade, T stage, N stage, pathology, and treatment may have clinical applications. As far as we are aware, our effort is the first to develop a special nomogram derived from LASSO to identify certain mGAC patients who may benefit from PTR.

Evidence supported primary tumor operation in patients with different stage IV malignancies [17–19]. It was indicated that removing the primary tumor can decrease the tumor load and eliminate the source of new metastases [20]. Previous studies revealed that patients with metastatic GC could obtain survival benefits from PTR more than non-operated cases [21–23]. Yeluri et al. indicated that the metastatic GC patients with PTR presented better postoperative survival than non-operation patients [24]. Huang et al. demonstrated longer OS of PTR-patients than those without resection (10.2 vs.4.5 months, P < 0.001) [25]. A Dutch study observed that primary tumor resection could improve prognosis in incurable GC patients, particularly for those with oligometastases and under 70 years old [21]. Leonardo et al. investigated patients with metastatic GC in the GIRCC database, finding that these patients could benefit from PTR after chemotherapy [26]. Even though the REGATTA research failed to present the benefit of PTR in advanced GC patients, it had intrinsic defects and generated other controversial results. For instance, only 13.8% of cardia tumors were found in the current SEER dataset in the PTR group, compared to nearly one-third of cancers in the upper part of the stomach in the PTR plus chemotherapy group. This might partly interpret the disappointing outcomes of REGATTA. However, it was clear that a specific population of mGAC could benefit from PTR. In our study, 307 patients in the PTR group obtained prolonged OS compared with the median OS in the non-PTR group, which was regarded as the beneficiary population. The PTR group’s improved survival can be attributed in part to a reduction in digestive tract blockage, tumor-related hemorrhages, and perforation [6–8]. Besides, circulating tumor cells (CTCs), the cancer cells in the bloodstream disseminated from the initial tumor location, have become a research hotspot in medical research [27]. It has been confirmed that the reduction in tumor load was associated with better survival in some malignancies [28, 29]. A recent study on CTCs indicated that PTR had a positive effect because the OS was considerably longer for patients who didn’t have CTCs than for those who had [30].

Gender, grade, T stage, N stage, pathology, and chemotherapy were the main prognostic factors in our visible nomogram, which could give specific estimations of whether mGAC patients would benefit from the removal of the primary tumor. In the earlier investigation, it was confirmed that these variables were connected to prognosis. Ma et al. constructed a predictive nomogram among mGAC patients after palliative gastrectomy, indicating age, tumor size, primary site, grade, T stage, N stage, the scope of gastrectomy, chemotherapy, and radiotherapy were significant factors [31]. Gao et al. found that tumor diameter, age, N stage, and grade were correlated with the prognosis of stage III/IV GC patients who underwent surgery [32]. According to the model, the early stage of T classification, N classification, and grade could promote the beneficial probability of the mGAC patients after PTR. The reason could be that individual heterogeneity existed in the mGAC patients, earlier T stage, N stage, and grade signified slow local tumor progression, although they are all stage IV patients. So mGAC patients could gain better survival after resection of the primary tumor. SRCC is a poorly differentiated subtype of GC with a dismal prognosis. It was founded that signet ring cells tended to transcoelomic metastasis more than other types of GC cells [33]. This study identified a similar conclusion to a previous study that a specific subgroup of metastatic gastric SRCC patients could achieve potential benefits from PTR [34]. The reason could be that PTR decreased the possibility of metastasis from the primary site. The male gender was an important factor influencing the occurrence of GC, which could be due to men’s unhealthy diet and lifestyle, such as smoking or abusing alcohol [35]. Besides, the analysis presented that chemotherapy combined with PTR could increase the benefit probability, which was in line with previous study [9]. The nomogram was evaluated in both groups. The results of AUCs (0.750 and 0.763) and calibration curves indicated the great predictive ability of the nomogram, which was better than the model in the former study (AUCs: 0.629 and 0.607) [31]. Moreover, the DCA curves indicated the good clinical value of the nomogram. Meanwhile, a relatively accurate and discriminating prognostic nomogram was developed to predict 3-, 6-, and 12-month CSS in mGAC patients, which included age, grade, T stage, N stage, surgery, and chemotherapy. And the model presented good predictive potential along with high credibility.

Surgeons frequently faced difficult issues regarding whether to perform primary tumor resection in patients who suffered metastatic gastric cancer, especially those with favorable clinical characteristics, including only one metastatic site, and those under 70 years old [21]. The predictive model could be a helpful method in this situation. Currently, there was no agreement regarding preoperative T and N classification for GC, but with the evolution of imaging methods, such as 3-Tesla magnetic resonance imaging and endoscopic ultrasonography, more precision assessment for tumor invasion and lymph node status might soon become a reality [36, 37]. Besides, the closer the treatment year is, the more scientific systemic treatment is available to these patients, which is also helpful to improve the effect of PTR. Since the patients in the study were enrolled from 2010 to 2015, we should have a more positive attitude toward PTR. With these effective methods, the predictive nomogram could help surgeons choose optimal candidates to perform palliative gastrectomy.

## Limitation

The research provided convincing results for identifying mGAC patients who could gain potential benefits from PTR. The current study did have several drawbacks, though, which warrant attention. First, crucial prognostic factors like postoperative comorbidities, chemotherapy regimen, removal of the metastatic tumor, and lymph node dissection type (D1 or D2) were absent from the SEER data repository. Second, because this was a retrospective analysis, selection bias was unavoidably introduced even if PSM was employed to reduce the heterogeneity across the groups. Therefore, additional multicenter prospective external validations were necessary.

## Conclusion

According to our research, mGAC patients in the PTR group had longer median OS than the non-PTR cohort. The high-performing benefit nomogram might help surgeons make decisions and choose the best PTR candidates. Additionally, the prognostic nomogram predicted each patient’s individual CSS probability at 3, 6, and 12 months, demonstrating good predictive ability. RCTs and additional study are needed to verify the conclusion.

## Supporting information

S1 Fig The mean difference between the two cohorts. (TIF)

S2 Fig Hazard ratios (HRs) for overall survival before PSM in the two groups are presented in forest plots. (TIF)

S3 Fig DCA curves of the benefit nomogram in the training (A) and validation (B) cohort. DCA curves of the prognostic nomogram in the training (C-E) and validation (F-H).
(TIF)

S4 Fig The calibration belt of the benefit nomogram. (TIF)

S5 Fig The ROC curves of subgroup analyses of different tumor locations. (A) cardia. (B) distal site.
(TIF)

S1 Table The basic characteristics of PTR patients in the training and validation group. (DOCX)
