# Supplementary figures and images for: Who are optimal candidates for primary tumor resection in patients with metastatic gastric adenocarcinoma? A population-based study
Source: PLoS One. 2024 Jan 24;19(1):e0292895. doi: 10.1371/journal.pone.0292895 (PMC10807831; doi:10.1371/journal.pone.0292895)

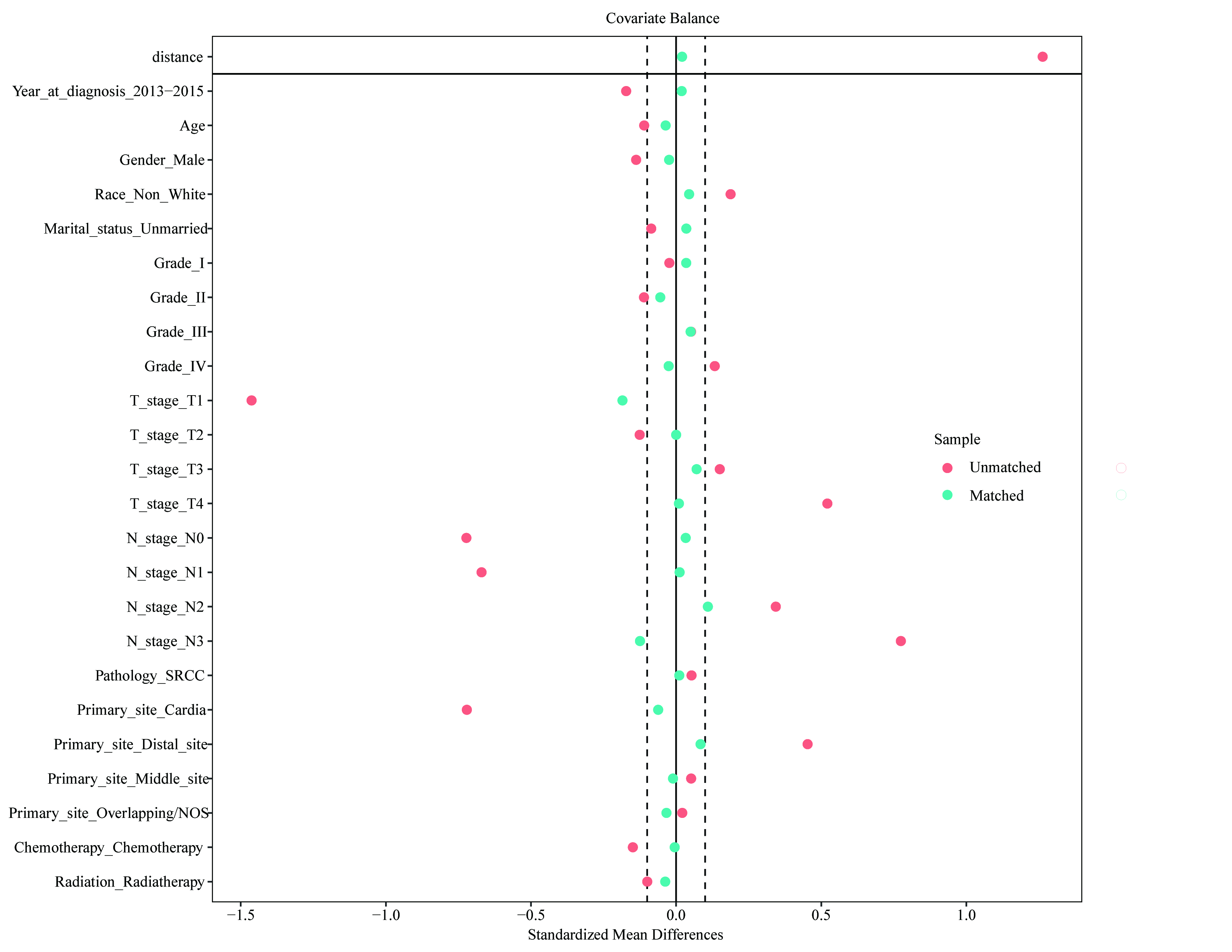

Supplement: S1 Fig — (TIF) [file pone.0292895.s001.tif]

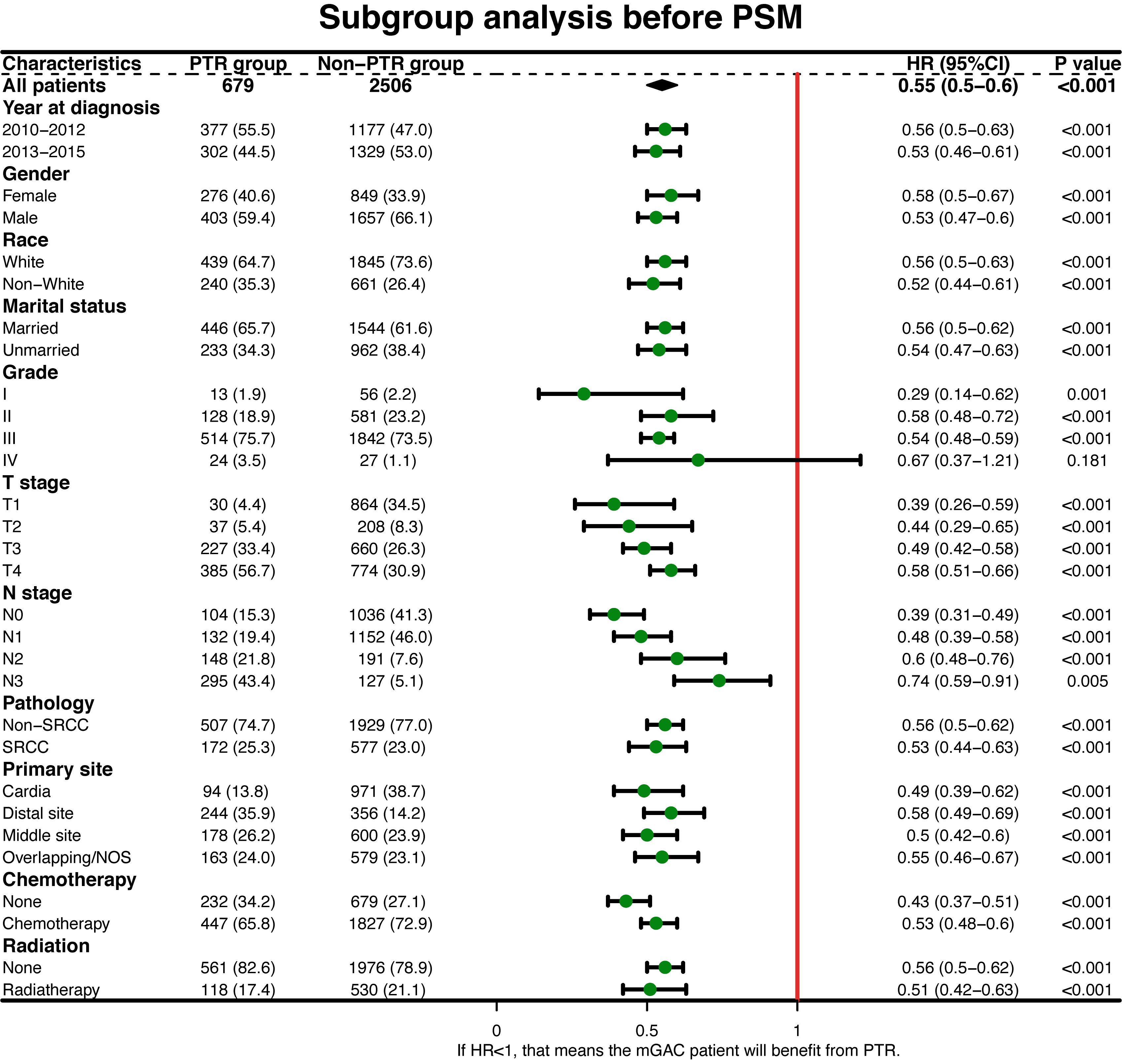

Supplement: S2 Fig — (TIF) [file pone.0292895.s002.tif]

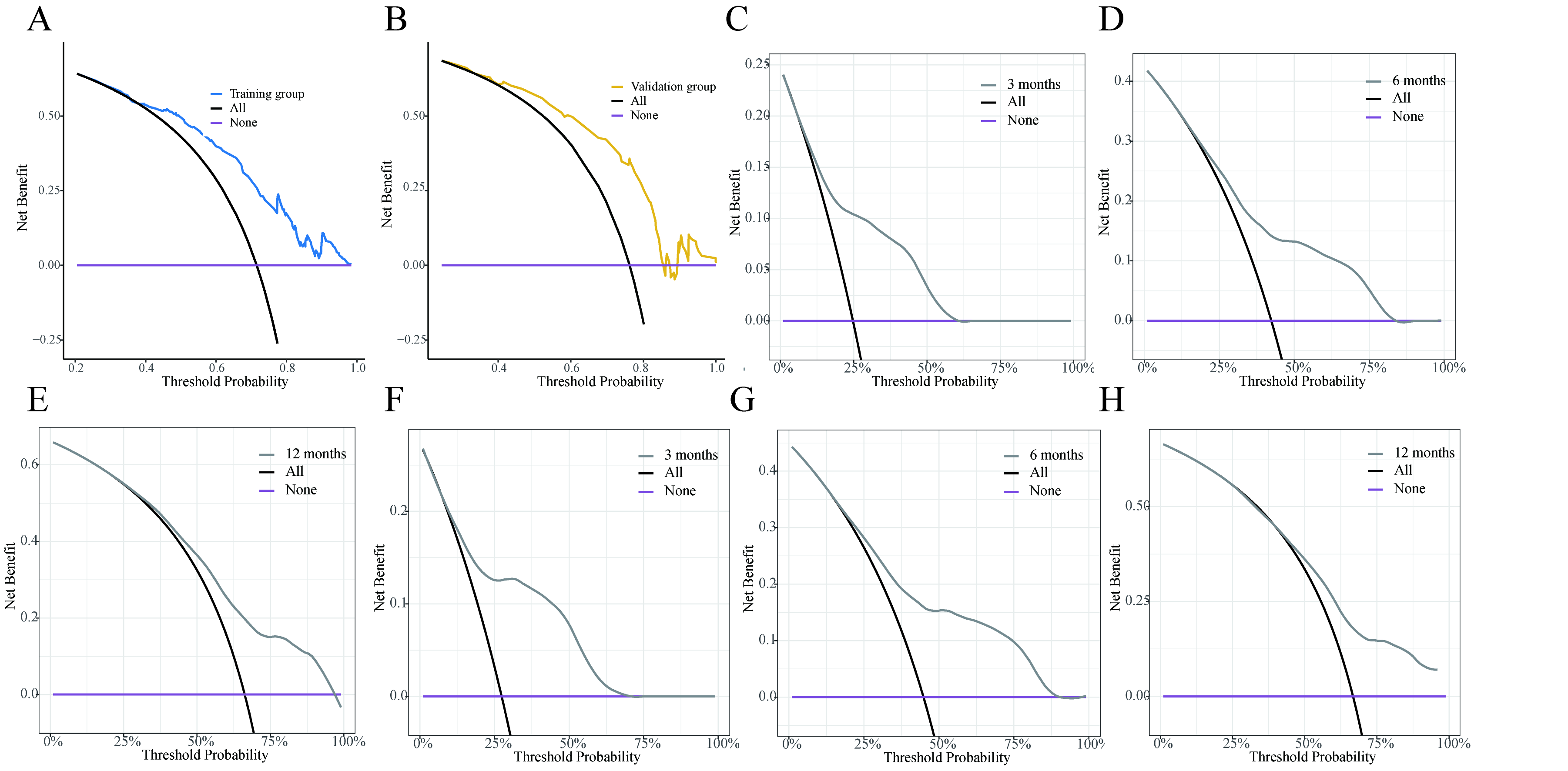

Supplement: S3 Fig — DCA curves of the benefit nomogram in the training (A) and validation (B) cohort. DCA curves of the prognostic nomogram in the training (C-E) and validation (F-H). (TIF) [file pone.0292895.s003.tif]

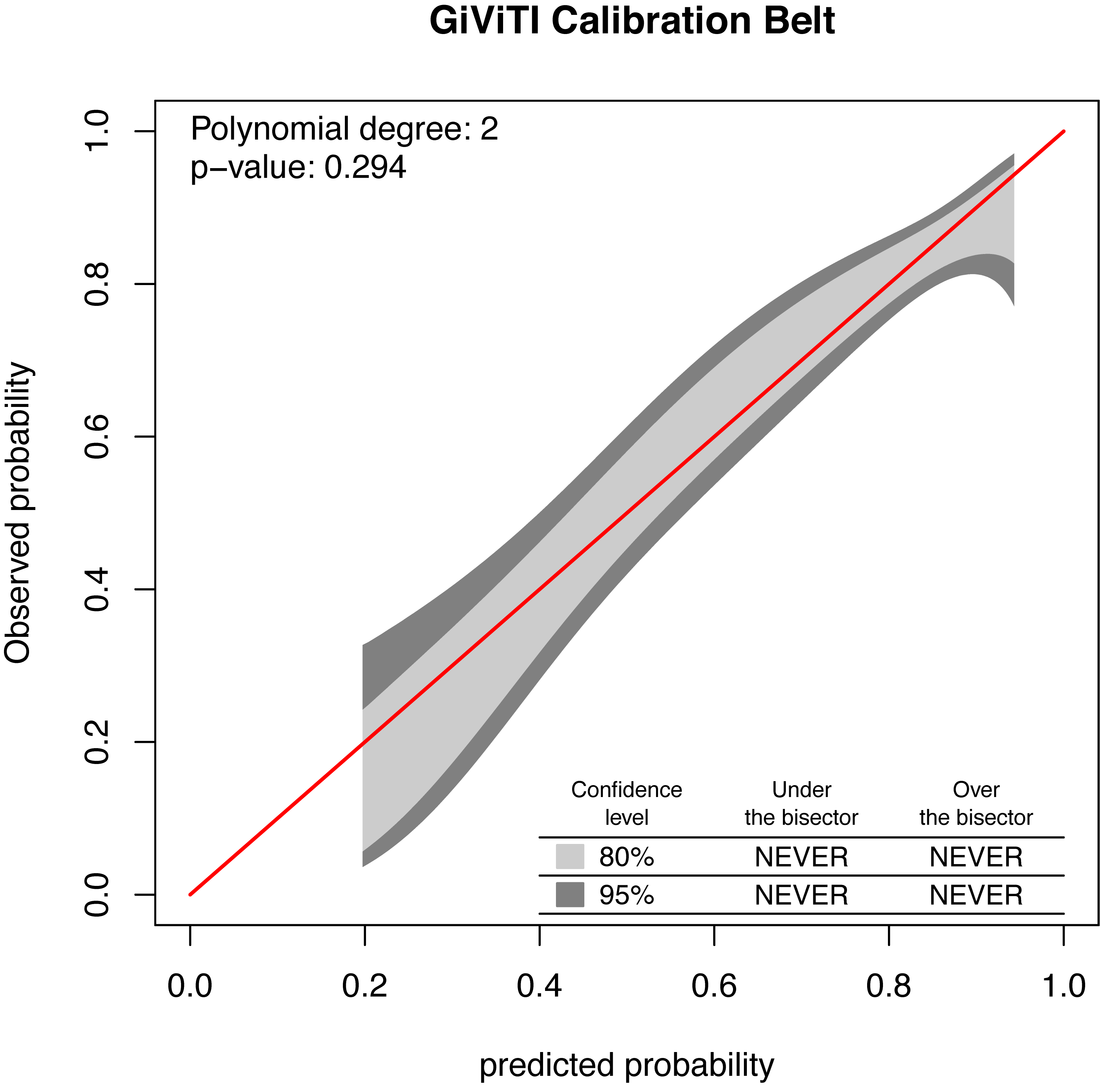

Supplement: S4 Fig — (TIF) [file pone.0292895.s004.tif]

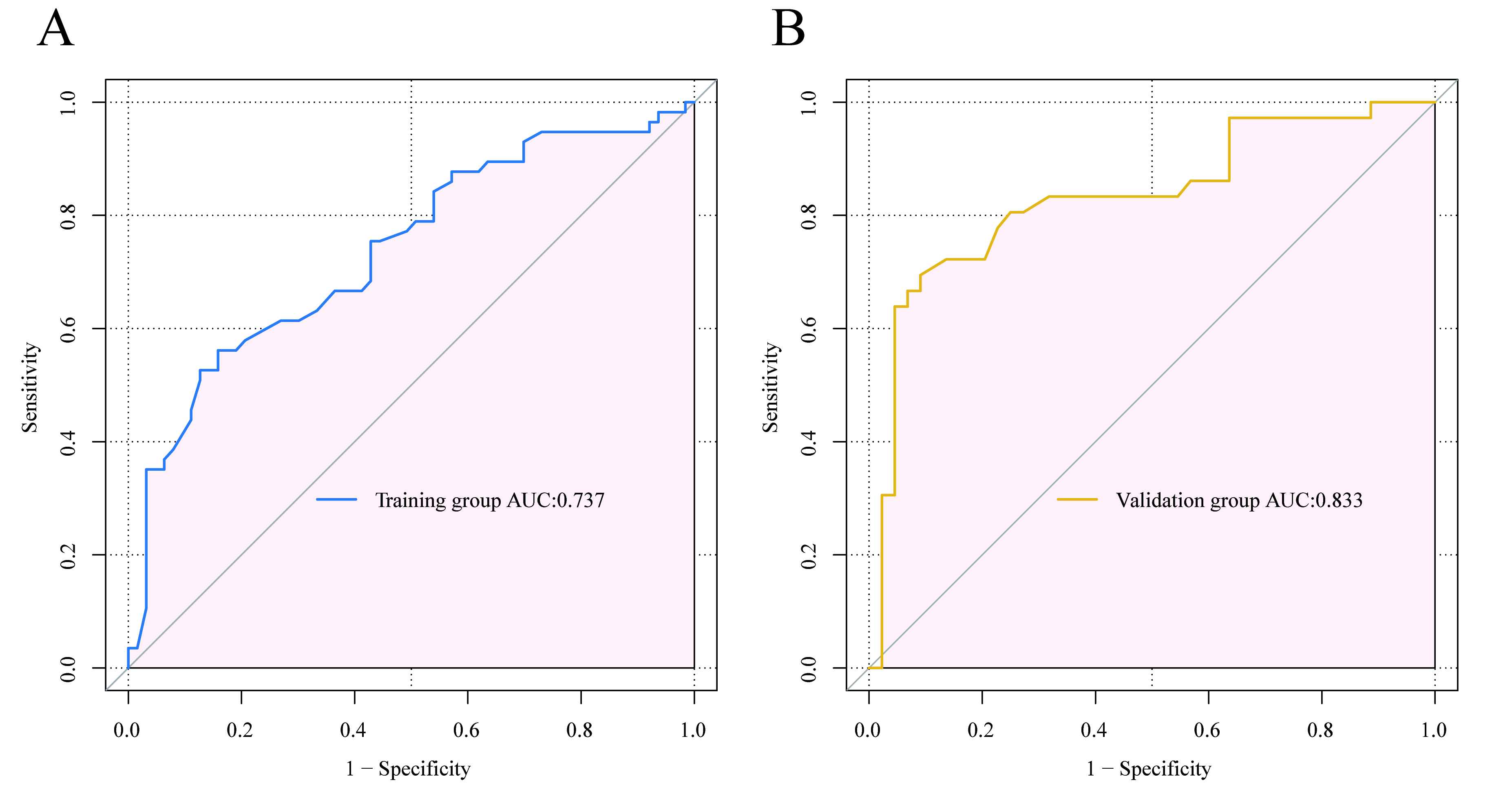

Supplement: S5 Fig — (A) cardia. (B) distal site. (TIF) [file pone.0292895.s005.tif]
