# Supplementary material for: Who are optimal candidates for primary tumor resection in patients with metastatic gastric adenocarcinoma? A population-based study
Source: PLoS One. 2024 Jan 24;19(1):e0292895. doi: 10.1371/journal.pone.0292895 (PMC10807831; doi:10.1371/journal.pone.0292895)
Supplement: S1 Table — (DOCX) [file pone.0292895.s006.docx]

| Table S1. The basic characteristics of PTR patients in the training and validation group | | | | |
| --- | --- | --- | --- | --- |
| Characteristics | All | Training | Validation | P value |
|  | N=421 | N=295 | N=126 |  |
| Year at diagnosis: |  |  |  | 0.709 |
| 2010-2012 | 208 (49.4%) | 148 (50.2%) | 60 (47.6%) |  |
| 2013-2015 | 213 (50.6%) | 147 (49.8%) | 66 (52.4%) |  |
| Age | 61.1 (14.6) | 60.6 (14.5) | 62.1 (14.9) | 0.365 |
| Gender: |  |  |  | 1 |
| Female | 159 (37.8%) | 111 (37.6%) | 48 (38.1%) |  |
| Male | 262 (62.2%) | 184 (62.4%) | 78 (61.9%) |  |
| Race: |  |  |  | 0.404 |
| Non-White | 141 (33.5%) | 103 (34.9%) | 38 (30.2%) |  |
| White | 280 (66.5%) | 192 (65.1%) | 88 (69.8%) |  |
| Marital status: |  |  |  | 1 |
| Married | 271 (64.4%) | 190 (64.4%) | 81 (64.3%) |  |
| Unmarried | 150 (35.6%) | 105 (35.6%) | 45 (35.7%) |  |
| Grade: |  |  |  | 0.378 |
| I | 10 (2.4%) | 6 (2.0%) | 4 (3.2%) |  |
| II | 85 (20.2%) | 55 (18.6%) | 30 (23.8%) |  |
| III | 318 (75.5%) | 227 (76.9%) | 91 (72.2%) |  |
| IV | 8 (1.9%) | 7 (2.4%) | 1 (0.8%) |  |
| T stage: |  |  |  | 0.684 |
| T1 | 30 (7.1%) | 20 (6.8%) | 10 (7.9%) |  |
| T2 | 31 (7.4%) | 19 (6.4%) | 12 (9.5%) |  |
| T3 | 166 (39.4%) | 118 (40.0%) | 48 (38.1%) |  |
| T4 | 194 (46.1%) | 138 (46.8%) | 56 (44.4%) |  |
| N stage: |  |  |  | 0.61 |
| N0 | 104 (24.7%) | 76 (25.8%) | 28 (22.2%) |  |
| N1 | 132 (31.4%) | 95 (32.2%) | 37 (29.4%) |  |
| N2 | 113 (26.8%) | 74 (25.1%) | 39 (31.0%) |  |
| N3 | 72 (17.1%) | 50 (16.9%) | 22 (17.5%) |  |
| Pathology: |  |  |  | 0.52 |
| Non-SRCC | 321 (76.2%) | 228 (77.3%) | 93 (73.8%) |  |
| SRCC | 100 (23.8%) | 67 (22.7%) | 33 (26.2%) |  |
| Primary site: |  |  |  | 0.268 |
| Cardia | 93 (22.1%) | 70 (23.7%) | 23 (18.3%) |  |
| Distal site | 118 (28.0%) | 87 (29.5%) | 31 (24.6%) |  |
| Middle site | 105 (24.9%) | 68 (23.1%) | 37 (29.4%) |  |
| Overlapping/NOS | 105 (24.9%) | 70 (23.7%) | 35 (27.8%) |  |
| Chemotherapy: |  |  |  | 0.909 |
| Chemotherapy | 294 (69.8%) | 207 (70.2%) | 87 (69.0%) |  |
| None | 127 (30.2%) | 88 (29.8%) | 39 (31.0%) |  |
| Radiation: |  |  |  | 0.662 |
| None | 337 (80.0%) | 234 (79.3%) | 103 (81.7%) |  |
| Radiatherapy | 84 (20.0%) | 61 (20.7%) | 23 (18.3%) |  |
| Metastatic site(s) |  |  |  | 0.521 |
| Distant lymph nodes | 71 (16.9%) | 49 (16.6%) | 22 (17.5%) |  |
| Viscera | 294 (69.8%) | 203 (68.8%) | 89 (70.6%) |  |
| Viscera plus distant lymph nodes | 29 (6.9%) | 17 (5.8%) | 9 (7.1%) |  |
| Distant metastasis, NOS | 27 (6.4%) | 26 (8.8%) | 6 (4.8%) |  |
| Benefit: |  |  |  | 0.386 |
| Benefit | 307 (72.9%) | 211 (71.5%) | 96 (76.2%) |  |
| Non-benefit | 114 (27.1%) | 84 (28.5%) | 30 (23.8%) |  |
